# Supplementary material for: Structural basis for substrate specificity and regulation of nucleotide sugar transporters in the lipid bilayer
Source: Nat Commun. 2019 Oct 11;10:4657. doi: 10.1038/s41467-019-12673-w (PMC6789118; doi:10.1038/s41467-019-12673-w)
Supplement: Supplementary file 1 — Supplementary Information [file 41467_2019_12673_MOESM1_ESM.pdf]

Structural basis for substrate specificity and regulation of nucleotide sugar transporters in the lipid bilayer.

Parker *et al*

Supplementary Figures 1-10 & Table 1

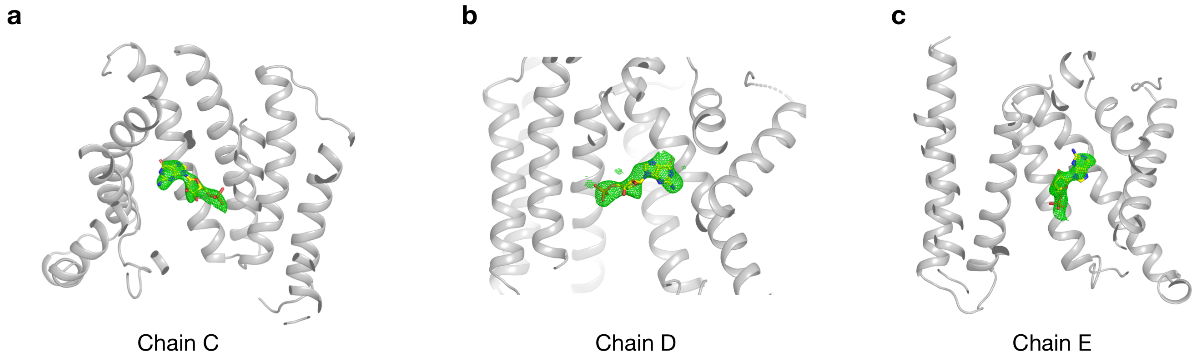

Supplementary Fig 1. Difference electron density maps for bound GMP. The mF_o-DF_c difference electron density observed for the GMP ligand in three of the eight transporters in the unit cell, contoured at 3 σ. GMP is shown as sticks and the electron density as green mesh.

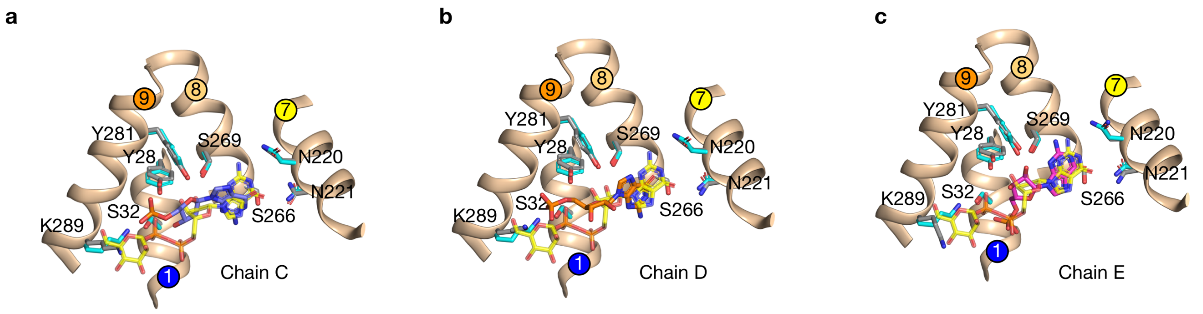

Supplementary Fig 2. Comparison of the position of bound GMP observed in the crystal structures with GDP-mannose. a, **b** & **c** show overlays of the respective GMP structures with GDP-mannose bound Vrg4 (PDB: 5OGK). Side chains from the GMP structures are shown in grey, while those from the GDP-mannose structure are shown in cyan. GMP and GDP-mannose are shown as sticks.

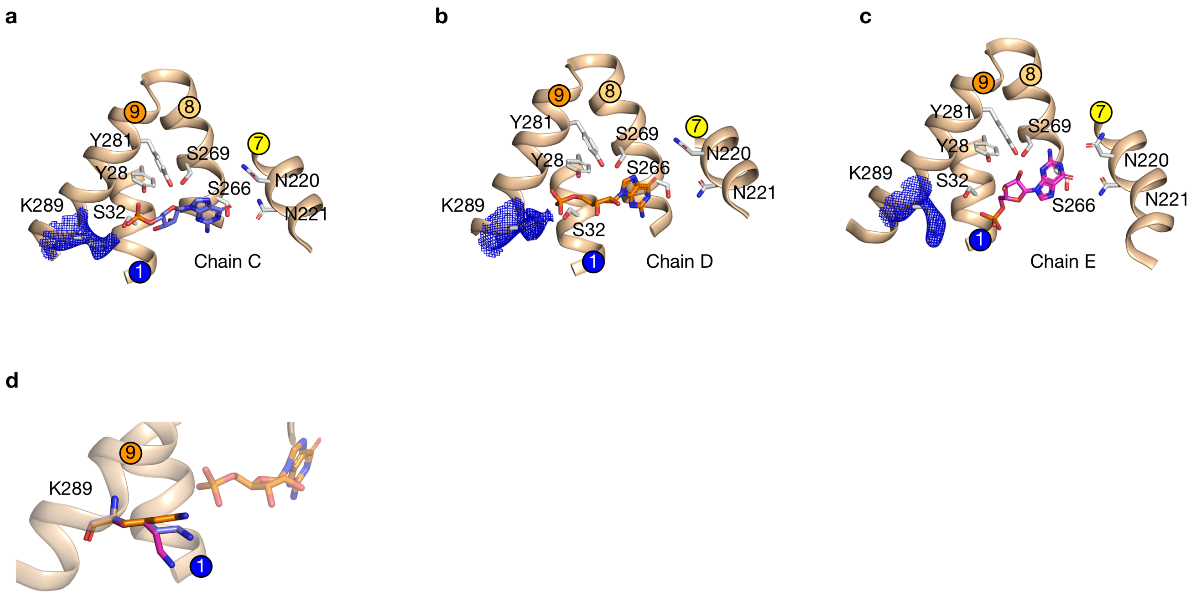

Supplementary Fig 3. Electron density maps showing different rotamer positions for K289. a, b & **c** The final refined 2FoFc electron density maps for the K289 side chain in three of the eight transporters in the unit cell is shown, contoured at 1 σ. **d** overlay of the three K289 side chain positions shown in a-c.

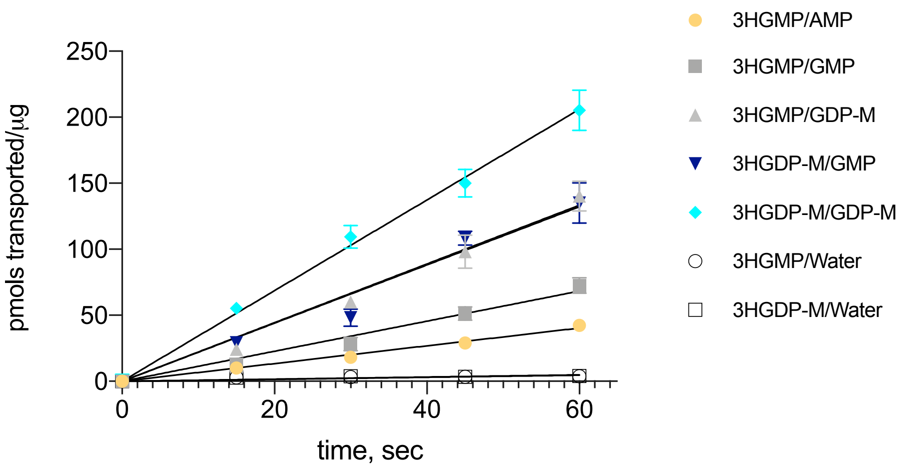

Supplementary Fig 4. Differing transport rates of Vrg4 observed with different ligand. The fastest rate of transport occurs when GDP-mannose is present on both sides of the liposome membrane and the slowest with AMP and GMP. n = 4 independent experiments, error bars s.d.

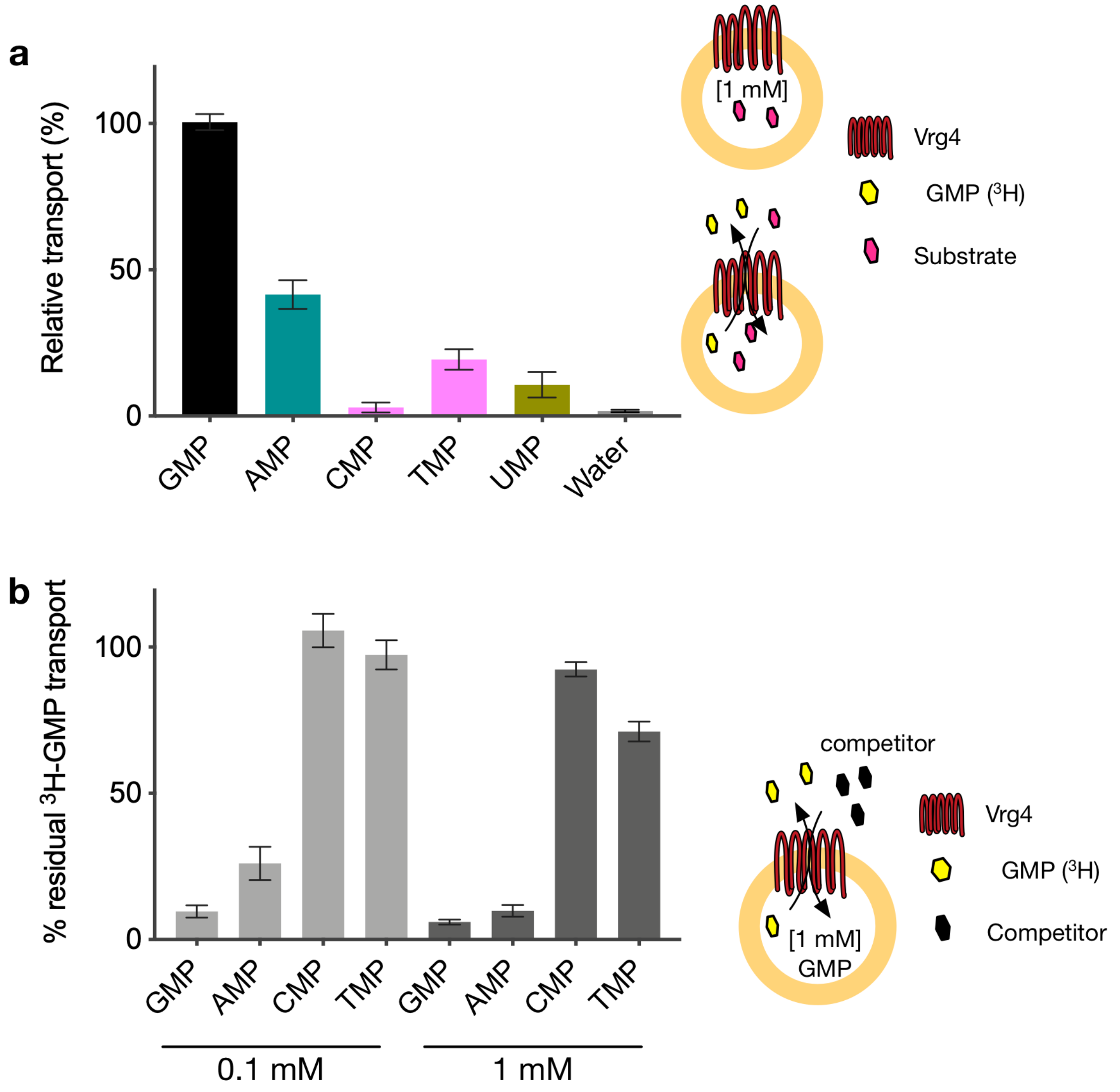

Supplementary Fig 5. Analysis of substrate specificity of Vrg4. a Vrg4 shows strict substrate specificity with respect to the nucleotide moiety, with a strong preference for purine bases for antiport and no transport observed when CMP is the counter transported molecule. **b** CMP cannot compete for GMP for transport, even at high concentrations (1mM).

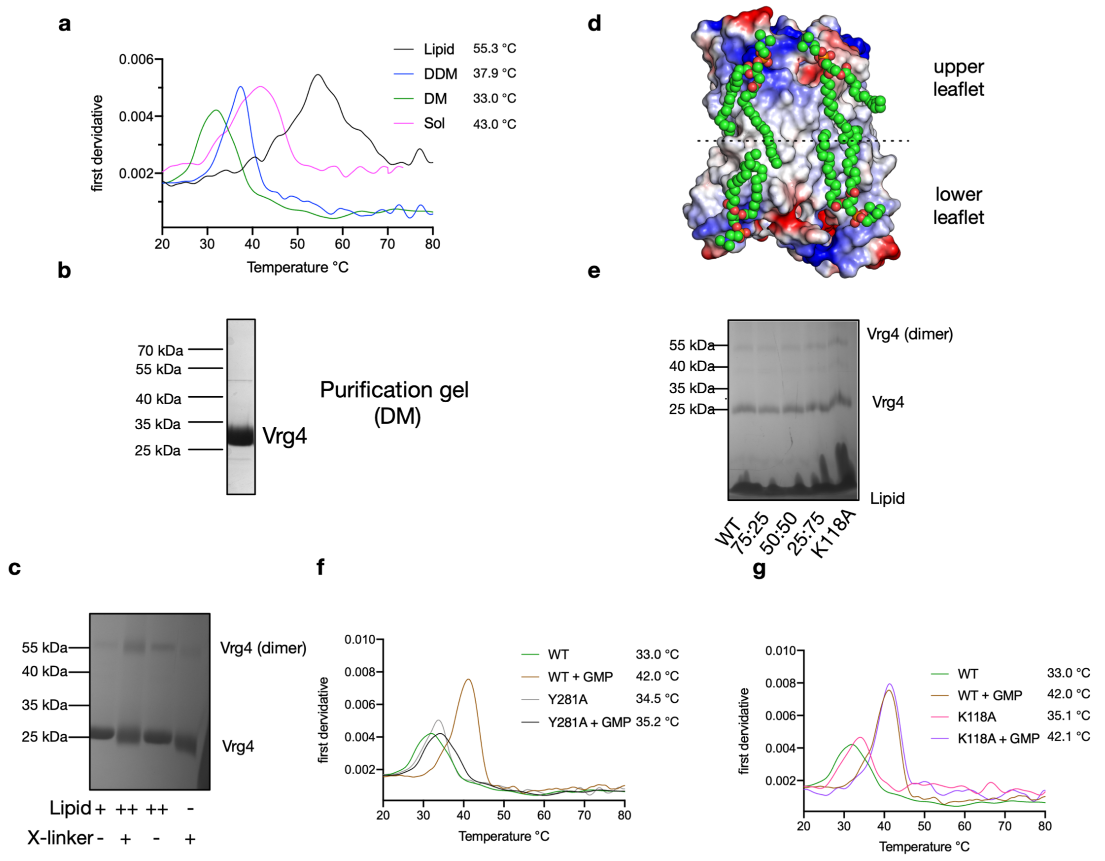

Supplementary Fig 6. Analysis of lipids on thermal stability of Vrg4. a Calculated melting temperature of Vrg4 in different environments; DDM - dodecylmaltoside, DM - decylmaltoside, lipid - reconstituted Vrg4 in yeast polar lipids. Melting temperature shown is mean calculated from 3 independent experiments. The data for the liposome experiment is more noisy due to a lower amount of protein being used, however the same amount of liposomes with no protein in them do not produce a signal in this experiment. However, liposomes containing Vrg4 which have been solubilised (Sol) in DDM (1 %) show a lower melting temperature than those in intact liposomes. **b** SDS-PAGE analysis of Vrg4 in the DM does not show the presence of a 'dimer' band. **c** Glutaraldehyde crosslinking showing that Vrg4 forms a dimer in the presence of lipid either 10 μg (+) or 20 μg (++). **d** View of the four DPPC lipids within the dimer interface of Vrg4 following ca. 30 ns simulation. **e** The Y281A variant of Vrg4 is not stabilised in the presence of 1 mM GMP unlike WT protein, indicating this variant is not able to bind GMP. N=3 independent experiments, data is the mean. **f** The K118A variant is stabilised to a similar extent as WT Vrg4 in the presence of 1 mM GMP,

indicating this variant is still able to bind GMP. N=3 independent experiments, data is the mean.

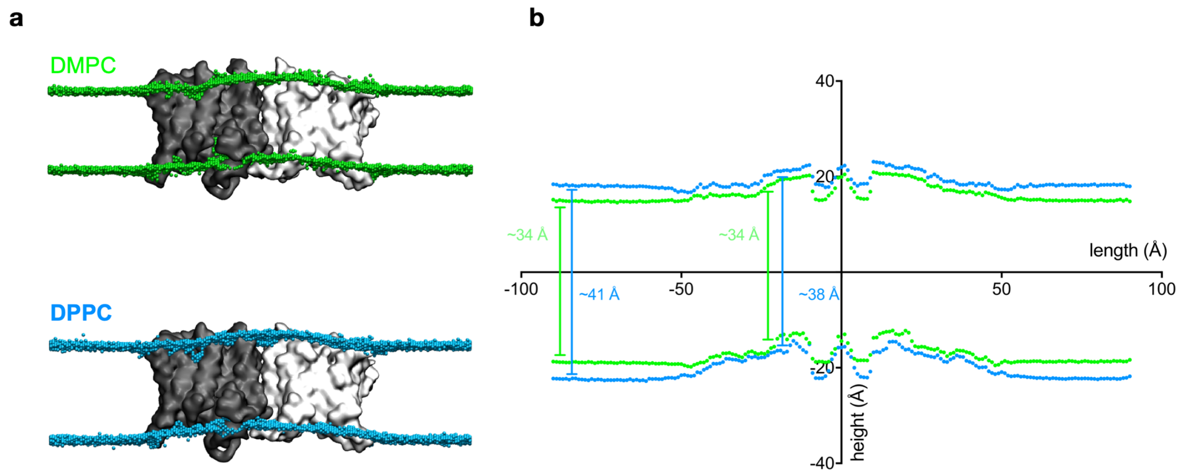

Supplementary Fig 7. Measuring the effect of Vrg4 on bilayer thickness. a Vrg4 shown as white and grey surface, and the average position of the lipid phosphate headgroups from CG simulation shown as green (DMPC) or blue (DPPC) beads. Beads more than ca. 10 Å from the protein to the front and back are removed for clarity. **b** Quantification of the data from panel A, with the coordinates of each point plotted as a function of height and length. In the bulk membrane, the DPPC (blue) is ca. 7 Å thicker than DMPC (green), whereas it thins considerably around Vrg4

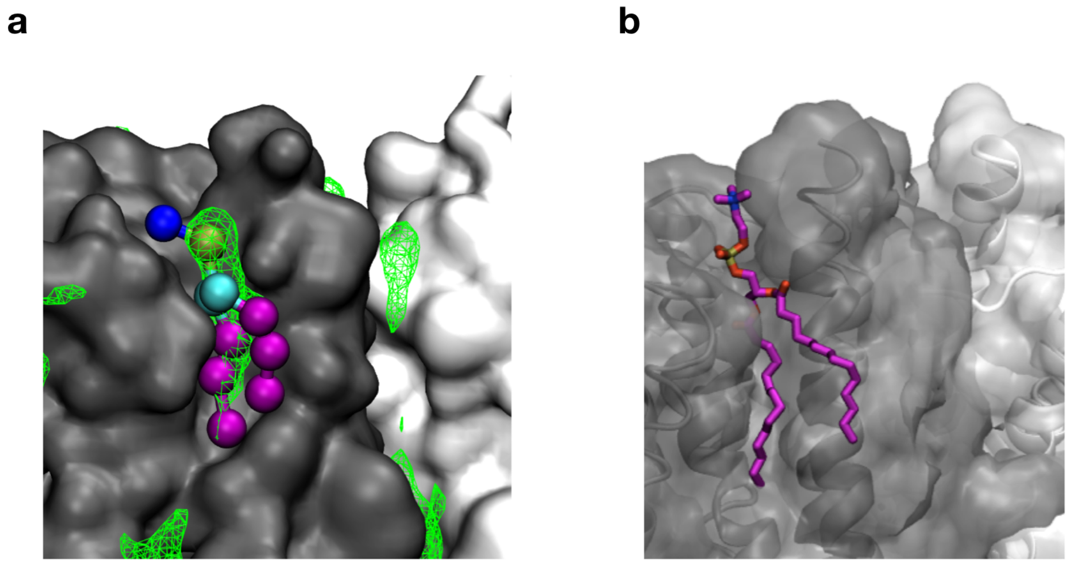

Supplementary Fig 8. Short chain lipids are observed within a second cavity in Vrg4. **a** View of DMPC lipid bound to the secondary lipid site of Vrg4 following ca. 15 μs of CG simulation. The computed DMPC densities (as shown in Figure 5b &c) is present as green mesh, with the protein shown as surface and the lipid shown as blue, tan, cyan and purple spheres. **b** A similar view to **a**, but following conversion of the system to an atomistic description. Here, the protein is shown as cartoon and transparent surface, and the lipid as blue, tan, red and purple sticks.

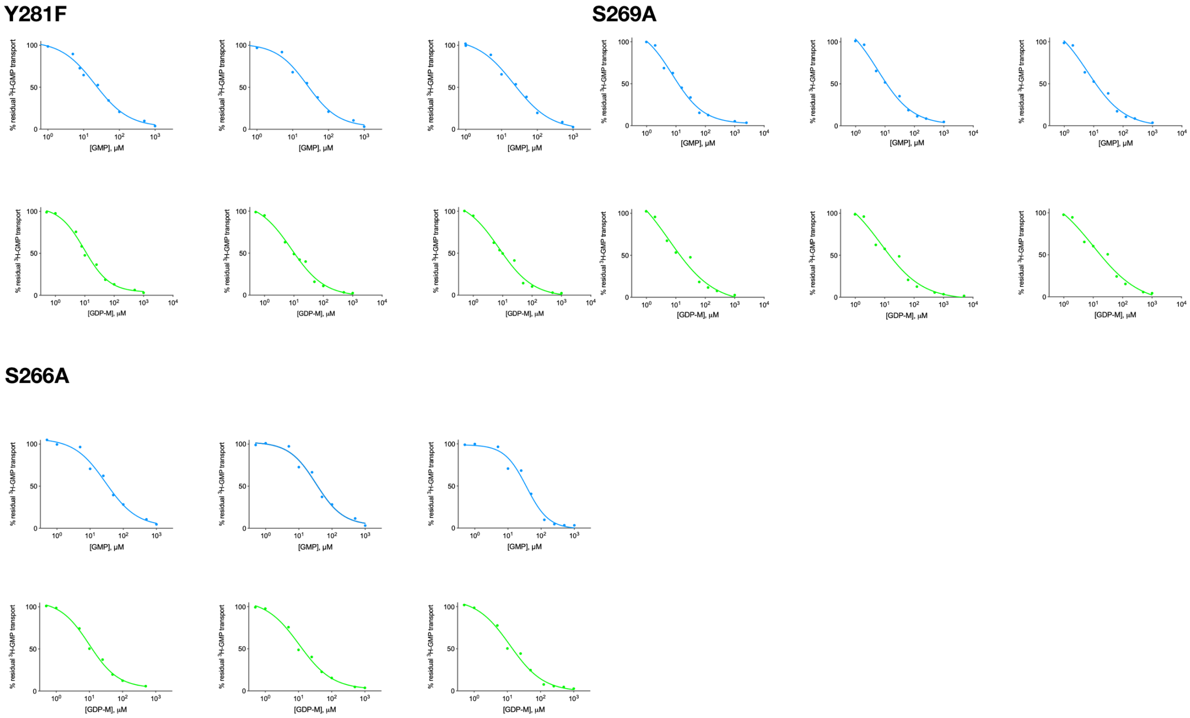

Supplementary Fig 9. Individual IC_{50} curves for GMP (blue) and GDP-mannose (green) calculated for Y281F, S266A and S269A variants of Vrg4.

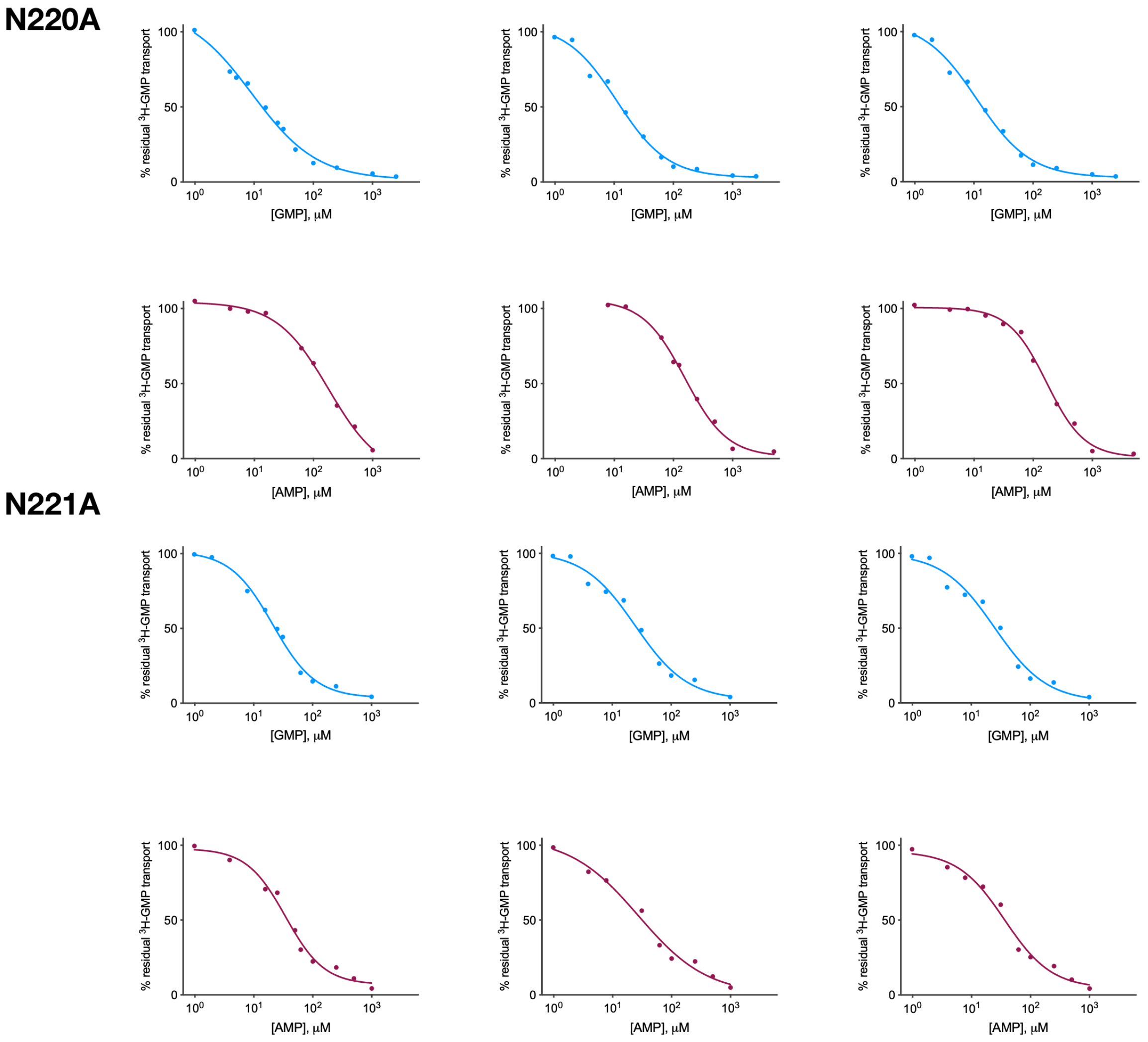

Supplementary Fig 10. Individual IC_{50} curves for GMP (blue) and AMP (purple) calculated for N220A and N221A variants of Vrg4.

Supplementary Table 1. Data Collection and Refinement Statistics.

	Vrg4-GMP PDB: 6QSK
**Data collection**	
Space group	P1
Cell dimensions	
a, *b*, *c* (Å)	45.71, 102.72, 181.25
α, β, γ (°)	89.93, 90.08, 90.19
Wavelength (Å)	0.980
Resolution (Å)[a]	49.43-3.39 (3.52-3.39)
R_{pim}	9.0 (61.4)
$I/\sigma I$	5.4 (1.2)
Completeness (%)	96.7 (92.3)
Multiplicity	2.0 (2.0)
CC1/2 (%)	99.7 (52.3)
**Refinement**	
Resolution (Å)	49.4-3.39
No. reflections	43971
R_{work}/R_{free}	25.3/31.9
Ramachandran favored	96.02
Ramachandran outliers	0.82
Molprobity score	2.01 (100th percentile)
Clashscore, all atoms	17.45 (97th percentile)

Supplementary Table 1. Data Collection and Refinement statistics. Values in parentheses are for the highest resolution shell.
